# Analysis and mapping of global scientific research on human monkeypox over the past 20 years

**DOI:** 10.14202/vetworld.2023.693-703

**Published:** 2023-04-06

**Authors:** Yasser Bustanji, Katia H. Abu Shihab, Waseem El-Huneidi, Mohammad H. Semreen, Eman Abu-Gharbieh, Karem H. Alzoubi, Mohammad A. Y. Alqudah, Ahmad Y. Abuhelwa, Eman Y. Abu-Rish, Hana Bajes, Khaled Obaideen, Islam Hamad, Nelson C. Soares, MoezAlIslam E. Faris

**Affiliations:** 1 Department of Basic Medical Sciences, College of Medicine, University of Sharjah, Sharjah, 27272, United Arab Emirates; 2 Research Institute of Medical and Health Sciences, University of Sharjah, Sharjah, 27272 United Arab Emirates; 3 Department of Biopharmaceutics and Clinical Pharmacy, School of Pharmacy, The University of Jordan, Amman, 11942, Jordan; 4 Department of Clinical Nutrition and Dietetics, University of Sharjah, Sharjah, 27272, United Arab Emirates; 5 Department of Medicinal Chemistry, College of Pharmacy, University of Sharjah, Sharjah, 27272, United Arab Emirates; 6 Department of Clinical Sciences, College of Medicine, University of Sharjah, Sharjah, 27272, United Arab Emirates; 7 Department of Pharmacy Practice and Pharmacotherapeutics, College of Pharmacy, University of Sharjah, Sharjah, 27272, United Arab Emirates; 8 Atlantic Cape Community College, Atlantic County, New Jersey, USA; 9 Department of Sustainable Energy and Power Systems Research Centre, RISE, University of Sharjah, P.O. Box 27272, Sharjah, United Arab Emirates; 10 Department of Pharmacy, Faculty of Health Sciences, American University of Madaba, Amman, Jordan; 11 Laboratory of Proteomics, Department of Human Genetics, National Institute of Health Doutor Ricardo Jorge (INSA), Av.a Padre Cruz, Lisbon, 1649-016, Portugal

**Keywords:** bibliometric study, coronavirus disease, epidemic, monkeypox, outbreak, smallpox, virus

## Abstract

**Background and Aim:**

Human monkeypox is an emerging global threat. Hundreds of publications were disseminated in the last few months. This study aimed to map, analyze, and evaluate the bibliometric indicators of the global monkeypox research output.

**Materials and Methods:**

All documents published in the past 20 years were retrieved using the Scopus database. Papers published in English and peer-reviewed journals were included. VOSviewer was used to create density and network visualization maps.

**Results:**

A total of 1725 published documents were retrieved. Of these, 53% were published in 2022. The average number of authors per document was 4.2. Authors from the USA were the most active and published about 42.1% of the total documents. International collaboration was evident between the USA and both UK and Congo. Keywords mapping identified the main research lines in this field that correlate monkeypox with public health, smallpox, vaccination, and antiviral treatment.

**Conclusion:**

This study analyzed and mapped the expanding field of monkeypox research across the world. The bibliometric analysis revealed that the United States has contributed greatly in terms of both individual researchers and academic institutions. There was less cooperation on a global scale than was anticipated. Fostering international cooperation is essential for countering this worldwide danger. Additional scientific research should be conducted to investigate the link between smallpox immunization and monkeypox epidemics.

## Introduction

At present, zoonotic viruses, such as human monkeypox, pose the most significant risk to global health, alarming pandemic outbreaks of emerging infectious diseases. Typically, zoonotic viruses can be transmit in both directions among animals and humans [1–3]. The Human monkeypox viruses, which belong to the orthopoxvirus genus and the Poxviridae family, have a large double-stranded DNA genome [4]. Typically, the infection is a self-limiting disease; classical symptoms can be relieved 14–28 days post-infection [5, 6]. However, the mortality rate fluctuates significantly from 1% to 11% [7]. Most registered deaths were young children who didn’t follow the standard vaccination programs for smallpox and/or immunocompromised patients, especially those diagnosed with human immunodeficiency virus (HIV) [8, 9]. The World Health Organization Research and Development (WHO R&D) has classified human monkeypox as an emerging disease (WHO R&D) [6].

In May 2022, the UK reported its first case of monkeypox virus infection [10]. Unfortunately, the number of detected cases progressively increased in a frightening pattern. Notably, the reported cases were distributed in different geographical countries worldwide. Despite being endemic to the African continent, over 80,000 cases have been detected in more than 110 countries within the last few months [11]. However, most of the cases are reported in the USA, European region and Canada, and countries of Latin America [12, 13]. The dramatic surge in monkey­pox-positive cases in non-endemic countries is a major reason for global concern as it highlights the disease’s potential for geographical spread and poses a significant global threat [6].

Given the cumulative volume of the literature on the emerging pandemic potential of monkeypox, it is essential to analyze and assess the global scientific literature on this hot topic. Bibliometric studies are investigations that map research tendencies and analytics on a certain topic. Bibliometric analyses are quantitative techniques that employ mathematical and statistical tools to measure the interrelationship and impact of publications within a given research area [14]. Bibliometric and visualization analyses have gained vast attractiveness in human and public health research in recent years [15], and this can be attributed, at least in part, to the growth, availability, suitability, and accessibility of many bibliometric and visualization software such as VOSviewer, CiteSpace, Bibliometrix, and Biblioshiny that are compatible with different research databases such as Scopus, Web of Science, Dimensions, Medline and PubMed [16–20].

The growing number of studies on a particular topic highlights the importance of bibliometric analyses. Through macro and micro analysis of thousands of published works, these studies allow for the identification of emerging trends and the intellectual framework of a given field [21]. Such analyses can provide a detailed overview of a huge number of disseminated literature and can professionally recognize leading studies, prolific authors, active journals, different collaboration patterns, contributing countries, and research institutions. Furthermore, these analyses can facilitate the exploration of current areas of interest and hot topics [22]. Not only, scholars and academics can get benefit from forming such investigations, but also, international organizations, funding sponsors, and policymakers can use bibliometric studies to explore the different scientific components of the existing literature [14].

Only a small number of bibliometric and visualization studies have examined the monkeypox emerging outbreak, as evidenced by references [23–25]. While these studies analyzed the global scientific output at an early stage following the first reported cases of monkeypox, the vast and rapidly increasing amount of literature on this topic necessitates an updated and comprehensive bibliometric analysis to gain a better understanding of the scientific discourse surrounding monkeypox.

This study aimed to dissect the global research output on human monkeypox literature published in Scopus-indexed peer-reviewed journals over the past 20 years (2002–2022). VOSview, Biblioshiny, and Microsoft Excel were used to retrieve many bibliometric indicators, including the total number of publications, countries’ contributions, collaboration patterns, most active journals, and publishing authors. Moreover, the author’s keyword and all keyword occurrence analyses were carried out to clarify the trends and hotspots of monkeypox research [26].

## Materials and Methods

### Ethical approval

No consent or ethical approval is required for this study.

### Study period and location

Data were retrieved on November 8, 2022 at College of Medicine, University of Sharjah. Sharjah, UAE.

### Search plan and refine the retrieved documents

All global research outputs published, over the past 20 years, about monkeypox in the Scopus database were retrieved and analyzed by bibliometric methods. Data were retrieved on November 8, 2022, using the following search term “monkeypox” OR “monkey pox.” All documents containing these search terms in the title, abstract, or keywords were retrieved. The search was also limited to the period from 2002 to 2022. The results were restricted to studies published in English and peer-reviewed journals. Books, book chapters, and conferences were excluded from the study.

### Data export

Retrieved documents were exported into CSV format for further data processing. Scopus Website and Microsoft Office Excel 2016 (Microsoft Corporation, Redmond, WA, USA), were used to analyze some of the bibliometric information such as research areas and journals. Geographical distribution of contributing countries (Geomap was created by Datawrapper (https://www.datawrapper.de).

### Bibliometric analysis and visualization

The latest version of the visualization of similarities (VOSviewer 1.6.18) (www.vosviewer.com) was used to map, and analyze collaborations, keywords, and citations in the retrieved documents [16–18]. The VOSviewer mapping method was used to create visualization networks and cluster analysis for countries’ collaboration, author-author collaboration, and author keywords. A density map of all keywords network was also generated. Further analyses of author keywords were performed using Biblioshiny software (Bibliometrix package, https://www.bibliometrix.org) [20]. It was used to analyze the author’s keywords trends over the studied years and prediction themes and hotspots of the current research.

In the analyses of the author’s bibliometric indicators, an inspection of the author names and their initials was performed manually for highly active researchers and then a thesaurus file was used to merge all author names with different initials, as they recognized as two different authors, into a single name (Karem L.K., Karem L.). Similar procedures were performed for keywords and contributing countries analyses. In the keyword analyses, all synonyms or similar keywords were merged into a single word. VOSviewer and Biblioshiny allow the user to perform such manipulation. Names of contributing countries also were corrected during the analyses of the retrieved documents. In a few documents, the country name was replaced by a city name (UK, London), or may be written in a different style (United Kingdom, UK). The absolute research output for each country was retrieved and then standardized, for each country, by dividing each country’s research output by its gross domestic product (GDP) per 1000 capita [27, 28].

## Results

### Analysis of publications by year

**Figure-1:**
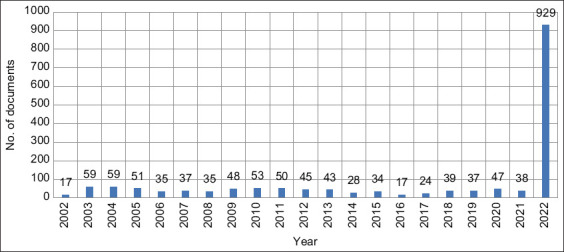
Growth of global research on monkeypox from 2002 to 2022.

According to our inclusion and exclusion criteria in searching for monkeypox in the past 20 years, 1725 documents were retrieved. Approximately 53% of these documents were published in 2022, while all other documents (47%) were published in the previous years (2002–2021) (Figure-1).

### Analysis of research subject areas

The top ten research areas in terms of the number of publications are listed in Table-1. Medicine as a research area was ranked number one with 1240 documents (72%). While Immunology and Microbiology Biochemistry, Genetics, and Molecular Biology field achieved about 29% of the total publications (n = 510). However, many articles were classified in more than one research area, and that’s why the total percentage exceeds 100%

**Table-1:** Top ten research areas of publications on human monkeypox.

Rank	Research area	Frequency	Percentage*
1	Medicine	1240	71.9
2	Immunology and Microbiology	510	29.6
3	Biochemistry, Genetics, and Molecular Biology	246	14.3
4	Pharmacology, Toxicology, and Pharmaceutics	111	6.4
5	Multidisciplinary	92	5.3
6	Agricultural and Biological Sciences	88	5.1
7	Veterinary	60	3.5
8	Environmental Science	58	3.4
9	Health Professions	49	2.8
10	Social Sciences	46	2.7

* Total exceeds 100% because of overlap in some documents among more subject areas

### Analysis of contributing journals

About 599 peer-reviewed journals, indexed in Scopus, have participated in publishing the 1725 retrieved documents. However, only 65 journals have published five documents and more. The top ten active journals that published about 22.6% of the disseminated literature are shown in Table-2. International Journal of Surgery was the most prolific journal with 51 (3.0%) publications, followed by Emerging Infectious Diseases and Travel Medicine and Infectious Disease with 49 publications for each. Eight of these top ten journals are ranked by Scopus as Q1.

**Table-2:** Top ten active journals publishing documents on monkeypox.

Journal Name	No. of publication	Percentage	Citations	Scopus percentile (Q)
International journal of surgery	51	3.0	74	99 (Q1)
Emerging infectious diseases	49	2.8	2202	92 (Q1)
Travel medicine and infectious disease	49	2.8	189	98 (Q1)
Viruses	42	2.4	630	76 (Q1)
Annals of medicine and surgery	40	2.3	59	43 (Q3)
Journal of medical virology	40	2.3	280	96 (Q1)
PLoS one	36	2.1	857	87 (Q1)
Journal of virology	34	2.0	1553	99 (Q1)
Virology	26	1.50	1030	63 (Q2)
Vaccine	23	1.30	687	97 (Q1)

### Analysis of articles

The retrieved articles have been cited 32,514 times, averaging 18.8 citations per document, with an *h-*index of 82. However, 940 documents have been cited <5 times. Table-3 shows the top ten cited documents [5, 29–36].

**Table-3:** Top ten cited documents on monkeypox research.

Rank	Authors	Title	Year	Citations	Normalized Citations/year	Journal
1	Siegel *et al.* [29]	2007 Guideline for Isolation Precautions: Preventing Transmission of Infectious Agents in Health Care Settings	2007	1366	91.1	American Journal of Infection Control
2	Galdiero *et al.* [30]	Silver nanoparticles as potential antiviral agents	2011	588	53.5	Molecules
3	Webby *et al.* [31]	Are We Ready for Pandemic Influenza?	2003	519	27.3	Science
4	Reed *et al.* [32]	The Detection of Monkeypox in Humans in the Western Hemisphere	2004	446	24.8	New England Journal of Medicine
5	Lloyd-Smith *et al.* [33]	Epidemie dynamics at the human-animal interface	2009	418	32.2	Science
6	Wolfe *et al.* [26]	Bushmeat hunting, deforestation, and prediction of zoonotic disease emergence	2005	384	22.6	Emerging Infectious Diseases
7	Karesh *et al.* [5]	Wildlife trade and global disease emergence	2005	371	21.8	Emerging Infectious Diseases
8	Antia *et al.* [34]	The role of evolution in the emergence of infectious diseases	2003	362	19.1	Nature
9	Rogers *et al.* [35]	A preliminary assessment of silver nanoparticle inhibition of monkeypox virus plaque formation	2008	329	23.5	Nanoscale Research Letters
10	Mcfadden [36]	Poxvirus tropism	2005	320	18.8	Nature Reviews Microbiology

### Analysis of authors

The total number of authors who contributed to the publication of the retrieved documents was 7251, with an average of 4.2 authors per document. There were 60 scholars who each contributed at least ten publications. The 10 most active authors are listed in Table-4. Damon, I.K., who is affiliated with National Center for Emerging and Zoonotic Infectious Diseases (NCEZID, Atlanta, United States) was the most prolific author with 70 published documents (4.6%). All authors in the list of top 10 prolific authors were affiliated with US institutions.

**Table-4:** Top ten authors publishing documents on monkeypox.

Rank	Authors	Documents	Country	Affiliation	Citations
1	Damon I.K.	70 (4.6)	USA	NCEZID	4150
2	Reynolds M.G.	50 (2.9)	USA	CDC	2106
3	Karem K.	45 (2.6)	USA	NCEZID	1757
4	Olson V.A.	35 (2.0)	USA	CDC	1950
5	Carroll D.S.	34 (2.0)	USA	CDC	1197
6	Mccollum A.M.	32 (1.9)	USA	CDC	981
7	Li Y.	29 (1.7)	USA	CDC	1981
8	Moss B.	22 (1.3)	USA	CDC	1039
9	Jahrling P.B.	21 (1.2)	USA	CDC	765
10	Hughes C.M.	20 (1.2)	USA	CDC	546

NCEZID=National Center for Emerging and Zoonotic Infectious Diseases, Atlanta, US, CDC=Centers for Disease Control and Prevention, Atlanta, US

### Active countries

**Figure-2:**
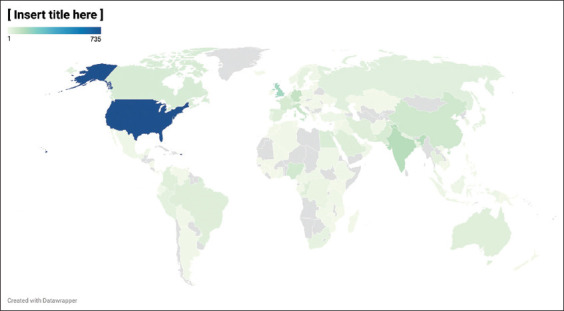
Geomap distrubution of conrtibuting countries in global monkeypox reserch output [Source: https://www.datawrapper.de].

In the Scopus database, the retrieved documents have been contributed by 122 countries, as depicted on the geomap in Figure-2. The top 10 countries in terms of the number of publications are presented in Table-5; however, the number of publications was normalized according to GDP. Table-5 shows the number of publications per GDP/1000 capita. The total number of citations for each country and the citation average for each publication are also shown in this table.

**Table-5:** Top ten active countries in publishing documents on human monkeypox.

Rank	Country	Number of publications (absolute research output)	% of total documents	Number of publications/GDP/1000 capita	Total citations	Citation/document
1	United States	735	42.6	10.5	20554	28.27
2	United Kingdom	168	9.7	3.51	2630	15.84
3	India	116	6.7	50.94	318	2.74
4	Germany	98	5.7	1.93	2871	29.30
5	Italy	79	4.6	2.22	1090	13.80
6	China	67	3.9	5.34	205	3.06
7	Nigeria	64	3.7	30.70	986	15.41
8	France	54	3.1	1.24	1041	19.28
9	Canada	53	3.0	1.00	1823	35.06
10	Pakistan	51	3.0	33.16	66	1.29

According to the number of publications, researchers from the USA have contributed the most to the field with 735 documents (42.6%) of total documents followed by the UK with 168 documents (9.7%). However, researchers from India have shown the highest publication rate when normalized to GDP/1000 capita with 51 documents/1000 capita when compared to the USA, which has a publication rate of 10.5 documents/1000 capita. It’s interesting to note that articles from Canada have the most scientific impact (35.1 citations/publication), followed by Germany. However, publications from India have shown the least scientific impact, with 2.5 citations per publication

### Active scientific institutions

The top ten active institutions are shown in Table-6. Centers for disease control and prevention (CDC) in the USA were the most active center in this field, followed by the U.S. Army Medical Research Institute of Infectious Diseases.

**Table-6:** Top 6 active organizations in publishing documents on human monkeypox.

Rank	Name	No. of documents	Percentage	Country
1	Centers for Disease Control and Prevention	141	8.2	USA
2	U.S. Army Medical Research Institute of Infectious Diseases	72	4.2	USA
3	National Institutes of Health	70	4.1	USA
4	National Institute of Allergy and Infectious Diseases	63	3.7	USA
5	National Center for Emerging and Zoonotic Infectious Diseases	49	2.8	USA
6	Harvard Medical School	31	1.8	USA
7	Emory University	30	1.7	USA
8	Organisation Mondiale de la Santé	28	1.6	USA
9	State Research Center of Virology and Biotechnology VECTOR	27	1.6	USA
10	Universidad Cientifica del Sur	25	1.4	Republic of Peru
10	Fundación Universitaria Autónoma de las Américas	25	1.4	Colombia

### Bibliometric mapping

#### Country-country collaboration

**Figure-3:**
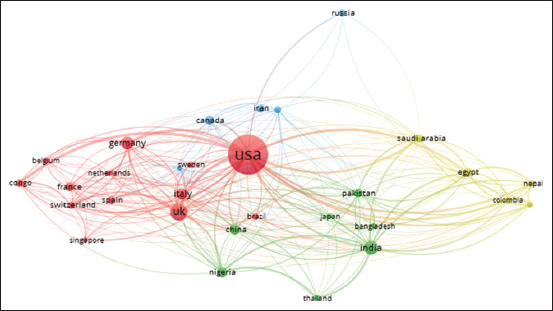
Network visualization map of counties’ research collaboration on monkeypox research. Only countries with a minimum of 15 publications were included.

International collaborations were mapped using VOS software and presented in a network visualization map (Figure-3). The countries on this map are depicted as spheres. Only 30 countries out of 122 met the minimum requirement of having 20 publications contributed from their country. Countries with the largest sphere size have more publications than countries with the smallest size. These counties were clustered into four groups indicating the strong collaboration between the countries of each group. These groups include group 1, which consists of 12 countries (red color): Belgium, Brazil, Congo, France, Germany, Italy, Netherlands, Singapore, Spain, Sweden, Switzerland, United Kingdom, and the United States. Group 2 consists of seven countries with green color, and these are Bangladesh, China, India, Japan, Nigeria, Pakistan, and Thailand). Group 3 consists of six countries (blue color) and these are Australia, Canada, Iran, Russia, and South Africa. Group 4 has five countries (yellow color) and these are Colombia, Egypt, Nepal, Peru, and Saudi Arabia. Authors from the same cluster of countries tend to publish together since they share common scholarly interests.

#### Author-author collaboration

**Figure-4:**
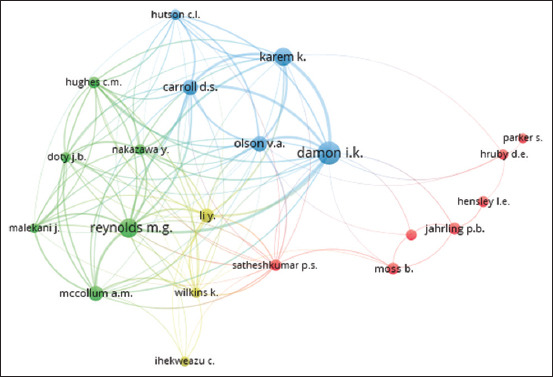
Network visualization map of author-author collaboration in the retrieved document. Only authors with a minimum of 15 publications were included. Twenty-one authors appeared on the map and they were clustered in 4 groups. Cluster 1 (red color): Hensley L.E., Hruby D.E., Jahrling P.B., Moss B., Parker S., Satheshkumar P.S., Shchelkunov S.N. Cluster 2 (green color): Doty J.B., Hughes C.M., Malekani J., Mccollum A.M., Nakazawa Y., Reynolds M.G., Cluster 3 blue color: Carroll D.S., Damon I.K., Hutson C.L., Karem K., Olson V.A. Cluster 4 yellow color: Ihekweazu C., Li Y., Wilkins K.

The author’s interactions were also studied. Collaborative authorship is depicted in Figure-4 as a network map (intellectual networking). If an author has <15 works published, they will not be considered. Only 25 authors of the 7251 contributors qualified in this respect. However, 21 authors appeared on the map and they were clustered into four groups indicated with different colors.

#### Analysis of author keywords

**Figure-5:**
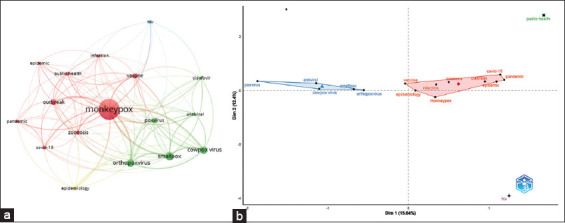
Analysis of the highest occurrence of author keywords. Keywords with a minimum occurrence of 20 times were included (a): Network visualization map (b) conceptual structure map.

Author keywords having a minimum occurrence of 20 times were considered in this study. The co-occurrence networks of author keywords are displayed in Figure-5a. The map contains 17 author keywords shown as circular nodes. Nodes are larger when they occur more frequently. Clusters of nodes with similar color schemes represent associations between them.

The author’s keywords of the retrieved literature on monkeypox were grouped into four clusters representing. The red-colored major cluster has a theme of monkeypox outbreaks, which is shown by the words: Coronavirus disease 2019 (COVID-19), epidemic, infection, monkeypox, outbreak, pandemic, public health, vaccine, and zoonosis. The second major green-colored cluster includes the theme of other similar infectious diseases, as it includes the words antiviral, cidofovir, cowpox, orthopoxvirus, pox virus, and smallpox. Meanwhile, another two groups appeared on the map as different clusters; HIV (blue) and epidemiology (yellow).

Author keywords were analyzed by Biblioshiny [37] to create a conceptual map using a minimum occurrence of 20 times (Figure-5b). Similar results in clustering groups were obtained from the two bibliometric software. These keywords were clustered around 4 different themes. The large one includes the monkeypox outbreak, while the second is concerned about poxvirus, smallpox, and other related viral infections cowpox, while the other two themes are related to HIV and public health.

**Figure-6:**
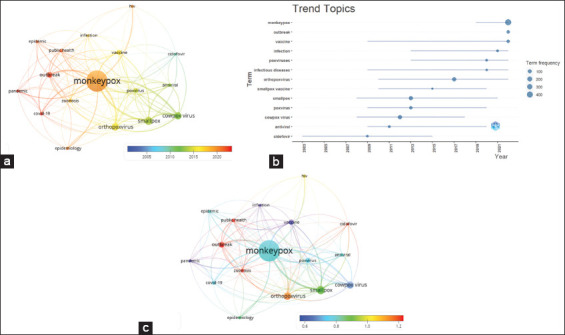
(a) Overlay visualization map of the highest occurrence of the author with average publication year overlay. (b) Author keywords trend the through the last 20 years (c): Overlay visualization map of the highest occurrence of author keywords, with an average normalized citations overlay.

Further analyses were performed on author keywords. Figure-6a shows an overlay of the keyword clusters normalized to the average publication year. The author’s keywords clusters were colored according to their publication years, that is, the red color indicates that these words were published recently. The cluster words (outbreak, public health, COVID-19, monkeypox, and pandemic) are the most recently researched in the years 2021–2022; meanwhile, the words small box, antiviral, and cidofovir are mostly researched in the years 211–2014. Moreover, author keyword analyses were also performed using Biblioshiny to map the research trends over the last 20 years (Figure-6b). Results show how these keywords, which reflect the topic trends, changed within the retrieved documents over the last 20 years. However, the results obtained from the two methods of investigation were similar. Monkeypox and outbreak were the trending topics in 2022, while smallpox was searched throw 2008–2021 and clustered in 2013. Research about cidofovir, the antiviral drug, was performed from 2003–2015 and peaked on this topic in 2009.

The retrieved documents with a particular author keyword received variable citations. Normalization of citations according to their time of publication could overcome the fact that older documents have more time to receive citations than more recent documents. The average normalized citations of the analyzed author keywords are shown in Figure-6c. The general terms of zoonosis, outbreak, public health, and orthopoxvirus received the highest normalized citations in comparison to the other author keywords.

#### Analysis of all keywords

**Figure-7:**
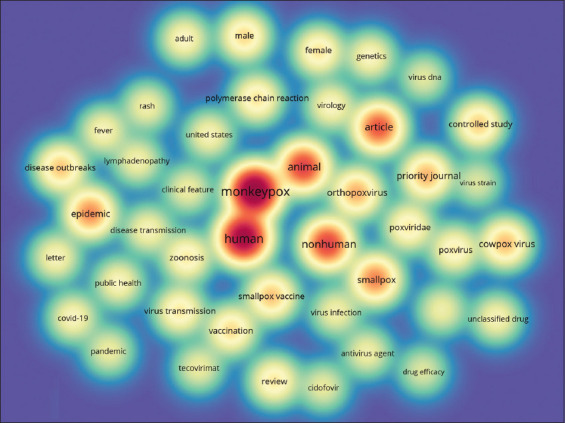
Visualization map of the highest occurrence of all keywords retrieved from title/abstract. Terms with a minimum occurrence of 100 times were included.

Co-occurrences of all keywords retrieved from titles/abstracts of the published documents were also analyzed. Figure-7 shows the density map visualization of all keywords co-occurrences. Terms with a minimum occurrence of 100 times were included in the study. Forty-four words were retrieved and presented in a density map. The intensity of the color is correlated to the number of occurrences. Hence, monkeypox, humans, animals, epidemic, smallpox, and disease outbreak were the most frequently encountered relevant keywords.

## Discussion

Human monkeypox, an uncommon infectious disease caused by the monkeypox virus, o is primarily seen in Africa. Recently, cases have emerged in at least 110 countries outside Africa [11, 24]. A prospective monkeypox outbreak, akin to the COVID-19 pandemic, is a health, political, and socioeconomic disaster that, if not controlled effectively, will have major negative consequences for the planet.

Despite the ongoing global impact of the COVID-19 pandemic, lessons and experiences garnered from the COVID-19 pandemic indicate that crisis and catastrophe management can be exceedingly difficult. Consequently, policymakers in the field of public health must enact essential policies and procedures for managing diverse threats during outbreaks. Consequently, acquiring knowledge about the pandemic is a crucial factor for the success of addressing its social, economic, and health impact.

Bibliometric analysis is a valuable method for understanding and analyzing large volumes of unstructured cumulative scientific knowledge and presenting these data in more structured pictures. In the current bibliometric study on monkeypox, we aimed to fill the knowledge gap and present the current global research output and trends in numerical forms. This will assess the scholars, health policymakers, and research institutions in focusing on how to address this potential threat.

Many databases are available for searching literature such as Scopus, Web of Science, Dimensions, PubMed, and Medline. The Scopus database was used in this study to retrieve all relevant documents. Scopus was selected for this study for several reasons; it is the largest abstract and citation database of peer-reviewed literature. Scopus Database provides basic and advanced search options that allow simple and comprehensive search inquiries. Scopus includes many analytical functions such as citation, subject, authors, countries analysis, and many other players. Data from Scopus can be easily manipulated and exported to analytical softwares or any other visualization program such as VOSviewer or Biblioshiny for further analyses and mapping. Scopus contains the highest number of journals compared to all other databases. Scopus-indexed journals are rigorous and systematically peered- reviewed and well-evaluated and are categorized according to several factors based on their CiteScore [27].

In this study, all documents regarding monkeypox through the past 20 years were retrieved. Documents were limited to the English language, and only peer-reviewed publications were included in the study.

The growth of the publications in the monkey pox field showed an abrupt increase in the last year 6 months since the infection emerged. Almost 54% of the total retrieved documents were published in 2022; noteworthy that the first documented monkeypox case was on May 2022, and the time of this search query was November 8, 2022. Approximately 929 documents were published in the period between May 2022 and November 2022 (Figure-1). This large number of documents disseminated in a relatively short period indicates global concern and interest in the potential for an outbreak, especially given that we are still experiencing a COVID outbreak for which the global research output on COVID-19 has been enormous and hundreds of thousands of documents have been published in the past 3 years.

The global health concern of the monkeypox outbreak could justify that the major these research areas where most of the documents were published were medicine (1240 articles, 71.9%), immunology and microbiology (510, 29.6%), biochemistry, genetics, and molecular (246, 14.3%) (Table-1).

The 1725 retrieved documents were published in 599 Scopus-indexed journals. The top ten active journals published about 22.6 % of all disseminated documents (Table-2). The current study showed relatively high citations (32514), with an average of 18.8 citations per document and a relatively high *h*-index of 82 for the published documents. This indicates the large number of interested readers in the subject. However, the *h*-index of the documents in the current study was less than that reported for literature on other hot topics such as antibiotic resistance [18], acquired immunodeficiency syndrome (AIDS)-related stigma [38], and HIV/AIDS-related medication adherence [38]. However, it is expected that the *h*-index of these documents will increase significantly in the coming month as the literature on monkeypox research is growing exponentially and these documents will receive more citations.

The high number of citations was further bolstered by the participation of high-impact publications in this field’s dissemination. Moreover, the global concern about the COVID-19 pandemic makes scientists, journals, health institutions, and research centers centralize and focus their efforts on monkeypox research in this relatively short period.

In terms of citations, *Emerging Infectious Diseases* has the highest number of citations. Interestingly, two articles from the top ten cited documents were published in this journal. The other eight highly cited documents were published in various journals from the top ten active journal lists. The highly cited documents were published from 2003 to 2011 (Table-3). Recent publications did not get enough citations to compete for these documents. The highly cited article by Siegel *et al*. [29], published by the American Journal of Infection Control, describes guidelines for isolation, precautions, and preventing transmission of infectious agents in healthcare settings, including monkeypox, and was published in 2007; moreover, this document gained the highest number of citations per year also. While the second highly cited document, either in terms of the absolute number of citations or when normalized to citation per year was the article published by Galdiero *et al*. [30], which was published in Molecules. This document describes the potential use of silver nanoparticles in the treatment of viral infections.

Based on an analysis of the geographic distribution of recovered papers, the United States published the most publications in absolute terms (Table-5 and Figure-2). Moreover, these documents were published by several American institutions. Eight of the top ten most active institutions were affiliated with the USA (Table-6). These centers have published approximately 510 documents, 29.6% of the total retrieved documents. Interestingly, the top ten active authors are affiliated with these institutions (Table-4); indeed, the top ten active researchers are affiliated with CDC and NCEZID in the USA. It’s expected that the USA, France, the UK, Germany, and Canada to be the leading countries in monkeypox research as they are countries with the highest numbers of monkeypox cases [11].

On the normalization of the number of retrieved documents/GDP/1000 capita, India, Pakistan, and Nigeria were the top active three countries, respectively. A recent study that looked at the patterns and trends in the scientific literature on the health problems caused by exposure to agricultural pesticides at work and home revealed similar country contribution trends, with the USA having more publications that have been disseminated overall than India but fewer when these publications are normalized for GDP per capita [27].

Analysis of the scientific impact of these countries, which can be inferred from the average citation per document (Table-5), revealed that documents retrieved from Canada have the highest scientific impact (35.1 citations/doc), followed by Germany (29.3 citations/document), and the USA approximately close to Germany with 28.3 citation/document. At the same time, China and India have scientific contributions of 3.1 and 2.5 citations/document, respectively. In this study, the retrieved documents gained 32514 citations. This high number indicates the importance of the subject and is also enhanced by the involvement of highly influential journals in publishing documents on this hot topic. This is evident as eight journals out of the top ten are classified as Q1 and the other two are Q2 journals (Table-2).

Countries with a high GDP spend significantly more on research and the construction of infrastructures essential for research than those with a low GDP. However, research and development spending has a beneficial effect on the number of absolute publications, the number of indexed journals, universities, and the *h*-index in various fields. It improves prospects for collaboration with national and international researchers and facilitates the use of existing resources, resulting in increased scientific output with a significant scientific effect [39, 40]. The scientific impact analysis of our study at this stage cannot be conclusive since almost 53% of disseminated documents were published in the past 6 months, which surely gained fewer citations compared to documents published years ago.

This research examined the collaborations between countries around the world (Figure-3). The largest nodes (the US, the UK, and India) have the greatest number of publications, while the smallest nodes (such as Sweden, Russia, and the Netherlands) have the fewest. This can be seen in Figure-3’s network visualization of countries’ research collaboration. When two countries are linked by a line, the thickness of the line indicates the extent to which their papers have been co-authored. The strongest research collaboration was depicted on the map by the thickest lines between the USA and Congo and the UK. On the other hand, the connections between Russia and other countries were the weakest, implying the least cooperation on a global scale.

Analysis of active authors in this field and their collaboration revealed that the top ten publishing authors are affiliated with USA institutions (CDC and NCEZID) (Table-4). However, they also collaborated with different authors in different worldwide institutions. Figure-4 shows a network visualization map of the author-author networking and collaboration. However, three clusters had five or more authors, each indicative of active collaborative research groups. In general, the minimum international collaboration of those authors can be seen as inferred from the thin connecting lines. While strong collaboration can be seen among the top active authors (green) who are affiliated with the two active centers in the USA (CDC and NCEZID). Analyses of both countries and authors’ collaborations, represented in Figures-3 and 4, indicate poor international interaction and collaboration on this hot issue.

Using VOSview and Biblioshiny to map the co-occurrences of author keywords in the retrieved literature on monkeypox, four overlapping theme clusters were identified. These clusters show the research focus and interest of the retrieved documents. The major research cluster is the one that associates monkeypox with outbreak issues, this is evident from the most occurrence words such as outbreak, infection, outbreak, and COVID-19. While the second most dominant theme focused on the nature of this virus and similar viruses, and their treatment and control; this appears clearly from the occurrence of words in this cluster such as antiviral, cidofovir, cowpox, poxvirus, and smallpox (Figure-5b). Monkeypox seems to be a disease that was discussed in earlier publications related to other similarly natured outbreaks such as cowpox and smallpox, through these words’ average publication year of 2013–2014 (Figure-6). These analyses also show how the topic trend is changed regarding the management of such viruses where the peak trend of “cidofovir,” an antiviral drug, was in 2009 has changed and reduced in the following years, while “vaccine” was found to have a higher frequency in the latest periods.

A density map depicting the highest frequency of all keywords extracted from the title/abstract shows the presence of 44 words (Figure-7). The most frequently encountered relevant keywords were monkeypox, human, animal, epidemic, smallpox, non-human, epidemic, and article. These words can show the special interest of researchers in the relationship between monkeypox with the absence of smallpox vaccination, as suggested by different studies [6]. The words of human, non-human, and animals also may imply that a large number of research was done on both human and animal subjects and the study of the non-human origin of orthopoxviruses [6].

This research has a few drawbacks. As we relied solely on Scopus to extract the documents, we cannot ignore the possibility that certain publications not indexed by Scopus may have published substantial research on this subject. We are also aware that not all publications were evaluated explicitly for their topical relevance. Moreover, only documents published in English were included in this study. Indeed, documents written in other languages may also contribute significantly to global research. Moreover, a small percentage of errors in the authors’ names and institutions may also affect the results of such studies.

## Conclusion

The current study analyzed and mapped the global research pattern of monkeypox research, an increasing issue of interest. After the initial diagnosis of monkeypox infection, the volume of research output grew dramatically. Analysis of the bibliometric indicators revealed the major contribution of American scholars and institutions. International collaboration was weaker than anticipated. Nonetheless, fostering international collaboration is essential to face this global threat. The association between smallpox immunization and outbreaks of monkeypox should be the focus of additional scientific studies.

## Authors’ Contributions

YB and WE: Conceptualization. KHAS, MAEF, NCS, and KO: Methodology. YB, EYA, HB, and AYA: Formal analysis. EA, MHS, and KHA: Investigation. MAYA and IH: Data curation. All authors have significantly contributed to the development and writing of this manuscript and have read, reviewed, and approved the final manuscript.

## Data Availability

Data included in article/supplementary materials/referenced in the article. Further data will be made available on request.
